# Acid Leaching Vermiculite: A Multi-Functional Solid Catalyst with a Strongly Electrostatic Field and Brönsted Acid for Depolymerization of Cellulose in Water

**DOI:** 10.3390/molecules27103149

**Published:** 2022-05-14

**Authors:** Xingtao Chen, Dongshen Tong, Zhi Fang, Zhenpeng Gao, Weihua Yu

**Affiliations:** 1 State Key Laboratory Breeding Base of Green Chemistry Synthesis Technology, Discipline of Industrial Catalysis, College of Chemical Engineering, Zhejiang University of Technology, Hangzhou 310014, China; 2111901180@zjut.edu.cn (X.C.); 2112001357@zjut.edu.cn (Z.F.); 2112001292@zjut.edu.cn (Z.G.); 2 Zhijiang College, Zhejiang University of Technology, Shaoxing 312030, China; ywh@zjut.edu.cn

**Keywords:** vermiculite, cellulose, hydrolysis, reducing sugars, layer charge

## Abstract

Vermiculite is a natural mineral. In this study, vermiculite and acid-activated vermiculite was used as a solid acid catalyst for the hydrolysis of cellulose in water. The catalysts were characterized by XRD, FT-IR, and BET. The effects of time, temperature, mass ratio and water amount on the reaction were investigated in the batch reactor. The results showed that the highest total reducing sugars (TRS) yield of 40.1% could be obtained on the vermiculite activated by 35 (wt)% H_2_SO_4_ with the mass ratio of catalyst to cellulose of 0.18 and water to cellulose of 16 at 478 K for 3.5 h. The acid-activated vermiculite was a stable catalyst through calcination at 628 K and the yield of TRS decreased to 36.2% after three times reuse. The results showed that the crystal structure of vermiculite was destroyed and the surface -OH groups increased after the acid treatment. However, the synergistic effect of a strongly electrostatic polarization and Brönsted acid was responsible for the efficient conversion of cellulose. The mechanism of cellulose hydrolysis on the acid-activated vermiculite was suggested. This work provides a promising strategy to design an efficient solid catalyst for the cellulose hydrolysis, and expands the use of vermiculite in a new field.

## 1. Introduction

Since the wide utilization of fossil resources has caused the global energy crisis and environmental pollution, the development of renewable resources is the alternative way to produce fuels and value-added chemicals [1,2,3]. Cellulose is the cheap, easily available, large reserve and non-edible lignocellulosic biomass in nature, and the catalytic conversion of biomass to produce liquid transportation fuels and fine chemicals is a promising route to decrease the environmental pollution and CO_2_ emissions caused by fossil fuel [4,5,6,7]. Cellulose is a linear long-chain polymer formed by D-glucopyranose units connected by β-1,4-glycosidic bonds [8]. One of the vital challenges in biomass conversion is the saccharification of cellulose into reducing sugars (RS, mainly glucose), which are water-soluble sugars and platform molecules that can be effectively converted into various chemical substances and fuels [2,6]. However, the intra- and inter-chain hydrogen bonds among cellulose make a high crystallinity structure, which is difficult for breaking the β-1,4-glycosidic linkages under mild conditions.

To address this issue, substantial efforts have been put toward the depolymerization of cellulose, including mineral acid hydrolysis, enzyme hydrolysis, supercritical water hydrolysis, and solid acid hydrolysis [5,7,8,9]. Among them, due to its easy separation and recovery, low cost, and mild reaction conditions, the process for the hydrolysis of cellulose into RS by solid catalysts has attracted more and more attention [6]. Up to now, many solid catalysts for cellulose hydrolysis have been studied, such as magnetic solid acid catalyst, sulfonated activated-carbon, heteropolyacid, Amberlyst 15 and so on [7,9,10]. However, the catalytic performance or the price of the solid catalyst is unsatisfactory.

Clay minerals are widely used as environmentally friendly catalysts for a number of reactions due to their specific structure and ubiquitous nature [11,12]. In our previous work, montmorillonite (Mt) was used as the catalyst for the hydrolysis of cellulose in water, and acid-activated Mt showed higher activity than that of ZSM-5. However, the activity and reusability of Mt-based catalyst was unsatisfactory for cellulose hydrolysis in water [13]. Among clay minerals, vermiculite (Vrm) is also the 2:1 type phyllosilicate, which is widely used in construction, agriculture and horticulture, metallurgical industry, animal husbandry, etc., [14,15,16,17,18,19,20]. In fact, although Vrm is in the same group of the smectites as Mt, Vrm has unique structure characteristics [21]. The crystal structure of Mt is dioctahedron, while Vrm is trioctahedron. The Vrm layer is composed of one Mg-O_4_(OH)_2_ octahedral sheet sandwiched between two opposing silicon-oxygen tetrahedral sheets. The layer charge mainly arises from the substitution of Al^3+^ for Si^4+^ in the tetrahedral sheet [14,22], which makes the higher layer charge density (0.6–0.9 eq./formula unit) than Mt (0.2–0.6 eq./formula unit) [14,23]. This unique structure characteristics significantly influences several physicochemical properties of clay minerals, such as higher cation exchange capacity, better thermal stability and thermal expansion [19,24]. A number of studies demonstrated that Vrm showed better properties than those of Mt, such as adsorption.

It is well-known that one of the effective methods for increasing the catalytic or adsorption activity of clay is the acid leaching. Such acid treatment can increase the amount of surface charges and the concentration of H^+^ in the layer due to the ion exchange and leaching out the structural ions (Al, Fe, Mg, etc.) [14,25,26,27,28]. Since the hydrolysis of cellulose is directly related to Brönsted acid sites in water, Vrm or acid-activated Vrm might be efficient for the cellulose conversion. Therefore, in the present work, modified Vrm obtained by acid leaching was used as the solid catalyst for the depolymerization of cellulose into reducing sugars in water. To the best of our knowledge, the application in the hydrolysis of cellulose has not yet been reported. This study presented that low-cost Vrm mineral was not only the efficient catalyst for the cellulose hydrolysis, but also high stability.

Highlights

1. Acid-activated vermiculite is the efficient catalyst for the hydrolysis of cellulose in water.
2. The regenerated vermiculite catalyst showed the high stability.
3. The electrostatic polarization had an important effect on the conversion of cellulose.
4. The synergistic effect of electrostatic polarization and Brönsted acid was responsible for the hydrolysis.

## 2. Experimental

### 2.1. Materials

The microcrystalline cellulose powder was purchased from the ShengDeLi Synthetic Leather Material Co., Ltd., Shanghai, China, and was obtained from cotton. The cellulose content was above 99 (wt)%. No physical or chemical pretreatments were used to treat the cellulose. Vermiculite was produced in 300 mesh from Lingshou County, Hebei Province, China. Amounts of 3,5-Dinitrosalicylic acid and methanol were purchased from Aladdin Chemicals Co., Ltd., Shanghai, China. Sulfuric acid (98%), hydrochloric acid (36%) and phosphoric acid (99%) were purchased from Aladdin Reagent Co. Ltd., Shanghai, China. All other chemicals were analytic purity and used without further purification.

### 2.2. Catalyst Preparation

The modified vermiculite samples were treated separately with three aqueous solutions of phosphoric acid, sulfuric acid, and hydrochloric acid. The acid-activated vermiculite was prepared as follows. 5/15/25/35/40 (wt)% H_2_SO_4_-activated Vrm was prepared by refluxing 90.0 g of 5 (wt)%, 15 (wt)%, 25 (wt)%, 35 (wt)% or 40 (wt)% H_2_SO_4_ and 10.0 g of Vrm for 6 h at 343 K in a 250 mL three-necked round bottom flask equipped with a condenser, a magnetic stirring bar and a thermometer. Then, the solid product was centrifuged, washed several times to neutral and dried at 353 K for 24 h. The products were designated as Vrm-HS5, Vrm-HS15, Vrm-HS25, Vrm-HS35, Vrm-HS40, respectively. Vrm activated by 35 (wt)% HCl or 35 (wt)% H_3_PO_4_ were prepared using the same method and the products were designated, respectively, as Vrm-HC35, Vrm-HP35.

### 2.3. Catalytic Conversion of Cellulose

All reactions were performed in a 30 mL stainless steel autoclave. In a typical experiment, a certain amount of cellulose powder, catalyst, and deionized water were added to the reactor, and then the reaction mixture was heated to a temperature (458–498 K) for a specified time (2.5–4.5 h). After the reaction, the solid-liquid separation of the reaction mixture was performed by centrifugation and the remaining residue was dried at 353 K for 12 h. The liquid products were quantitatively analyzed by 3,5-dinitrosalicylic acid (DNS method), and the absorbance at 520 nm was measured using an ultraviolet-visible spectrophotometer (UV-2500) to determine the total reducing sugar (TRS) yield [29,30,31]. The solubilization percent of cellulose and yield of TRS were calculated as follows:

(1) $$\mathrm{Solubilization}\;\left(\mathrm{wt}\%\right)=\left(1-\frac{\mathrm{Mass}\;\mathrm{of}\;\mathrm{the}\;\mathrm{recycled}\;\mathrm{solid}\;-\;\mathrm{Mass}\;\mathrm{of}\;\mathrm{the}\;\mathrm{catalyst}}{\mathrm{Mass}\;\mathrm{of}\;\mathrm{initial}\;\mathrm{cellulose}}\right)\times 100\%$$

(2) $$\mathrm{TRS}\;\mathrm{yield}\;\left(\mathrm{wt}\%\;\right)=\left(\frac{\mathrm{Concentration}\;\mathrm{of}\;\mathrm{product}\;\mathrm{RS}\;\times \;\mathrm{Volume}\;\mathrm{of}\;\mathrm{reaction}\;\mathrm{solution}}{\mathrm{Mass}\;\mathrm{of}\;\mathrm{initial}\;\mathrm{cellulose}}\right)\times 100\%$$

### 2.4. Characterization

X-ray diffraction (XRD) measurements were collected using a PANalytical X’Pert PRO diffractometer between 5° and 40° (2θ), a scan rate of 0.1/s, and copper Kα radiation (λ = 0.1541 nm). A Nicollet 6700 Fourier transform spectrometer was used to record the Fourier transform infrared spectrum between 4000 and 400 cm^−1^. The sample was dried at 383 K, mixed with KBr, and exposed to infrared light. The pellets were immediately measured after preparation under ambient conditions in the mid-infrared region. The spectra are the average results of 32 scans in the wavelength range of 4000 to 400 cm^−1^. The surface areas were determined by N_2_ adsorption at 77 K using a Micromeritics ASAP 2020 instrument. The samples were outgassed in vacuum for 12 h at 383 K prior to nitrogen adsorption. The surface area was calculated using the BET method based on adsorption data in the partial pressure (P/P_0_) range 0–1.0.

The measurement procedure of the hydroxyl group on the catalyst was as follows [30,31,32]: The catalyst sample (0.05 g) was treated with 0.01 mol/L sodium chloride solution (20 mL) under ultrasonic vibration at 303 K for 1 h. After centrifugation, the supernatant was titrated with 0.01 mol/L sodium hydroxide solution, using phenolphthalein as an indicator.

### 2.5. Catalyst Regeneration and Reusability Tests

The stability and reusability of the catalyst were tested under optimal conditions. After reaction, the catalyst was separated by centrifugation, washed with ethanol, and dried at 373 K. Additionally, the recycled solid was regenerated by calcination at 623 K for 4 h. Then the regenerated product was grounded and the first-time, second-time and third-time recycled catalysts were designated as Vrm-1st, Vrm-2nd and Vrm-3rd, respectively. The regenerated catalyst was replenished to make the initial weight identical to the first cycle and the catalytic performance was tested under the optimum conditions. The concentration of TRS was measured with the same method.

## 3. Results and Discussion

### 3.1. Characterization of Catalysts

**Figure 1:**
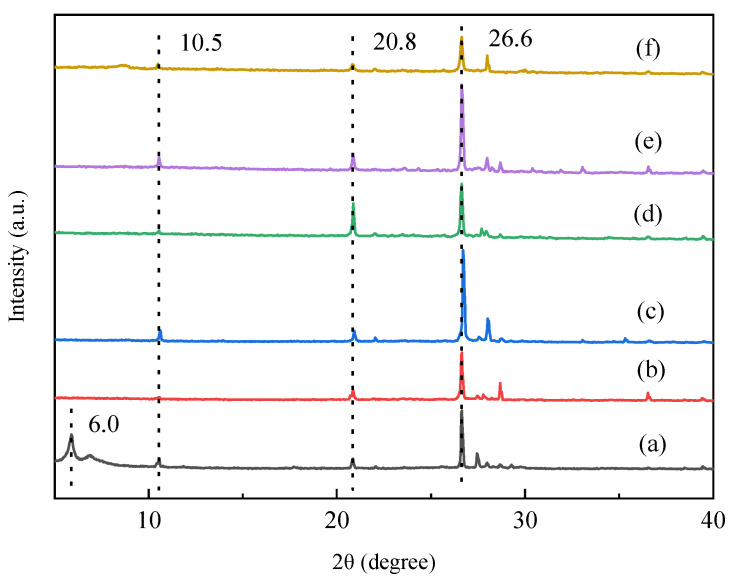
XRD patterns of Vrm (a) Vrm-HS15 (b) Vrm-HS35 (c) Vrm-HC35 (d) Vrm-HP35 (e) Vrm-3rd (f).

Figure 1 shows the XRD patterns of fresh and acid-activated vermiculite catalysts. According to Figure 1, the typical diffraction peaks of vermiculite at 2θ = 6.0° correspond to (002) crystal plane [17,18,24,33]. The diffraction peaks at 2θ = 20.8 and 26.6° are ascribed to the impurities (quartz). The peak at 2θ = 10.5° belongs to Ferrogedrite (JCPDS#31-0617). The typical diffraction peaks of Vrm almost disappeared after acid treatment, demonstrating that the crystal structure of Vrm was destroyed [34]. After regeneration, the typical diffraction of Vrm disappeared. This result demonstrated that the crystal structure of Vrm could be easily destroyed with the acid treatment and the crystal structure could not be recovered by calcination [16,25,34].

**Figure 2:**
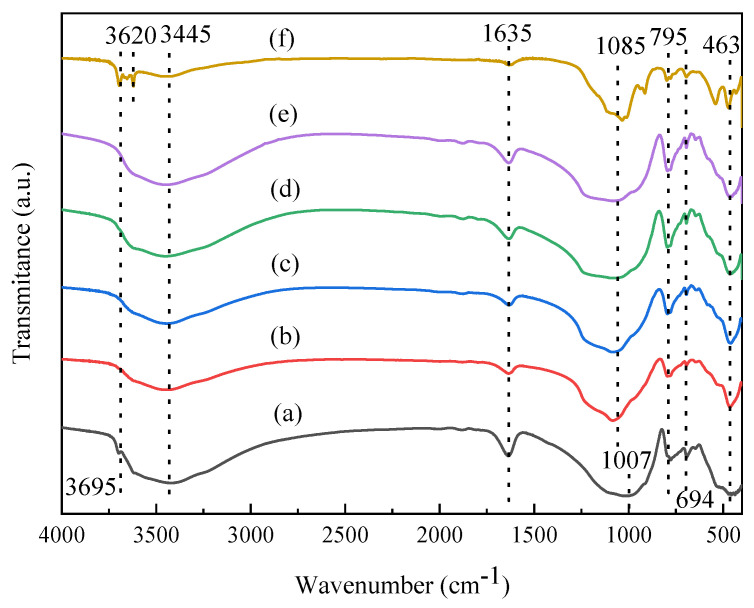
FT-IR spectra of Vrm (a) Vrm-HS15 (b) Vrm-HS35 (c) Vrm-HC35 (d) Vrm-HP35 (e) Vrm-3rd (f).

Figure 2 presents FTIR spectra of raw and acid-activated vermiculite catalysts. From Figure 2, the band observed at 3445 cm^−1^ is due to the adsorbed water on Vrm. The stretching vibration of -OH group appears at 3695 cm^−1^, which is coordinated to the octahedral layer [35,36]. The band at 1635 cm^−1^ is attributed to the bending vibration of water. The peak at 1007 cm^−1^ corresponds to the stretching vibration of Si-O-Si and Si-O-Al and the peak at 463 cm^−1^ is the Si-O-Mg bending vibration. Peaks at 795 cm^−1^ and 694 cm^−1^ are ascribed to the symmetric stretching vibration peak of amorphous silica (Si-O-Si) or Si-O-Al [24,25]. For the acid-activated Vrm, the shoulder peak at 3695 cm^−1^ and the peak at 463 cm^−1^ both became stronger. It might be that the layer structure of Vrm was destroyed and the octahedral layer was exposed. Moreover, the peak at 1007 cm^−1^ was shifted to 1085 cm^−1^, which should be attributed to the leaching of Al ions in the tetrahedral layer [17]. For the regenerated sample, there existed two strong bands at 3695 and 3620 cm^−1^, which are ascribed to -OH groups coordinated to the octahedral layer and Si-OH groups. It also presented that the octahedral layer was exposed and there had more surface -OH groups after regeneration. Therefore, from the FTIR results, it also showed that the layered structure of Vrm was destroyed after acid treatment and there appeared more -OH groups on the surface of Vrm.

**Figure 3:**
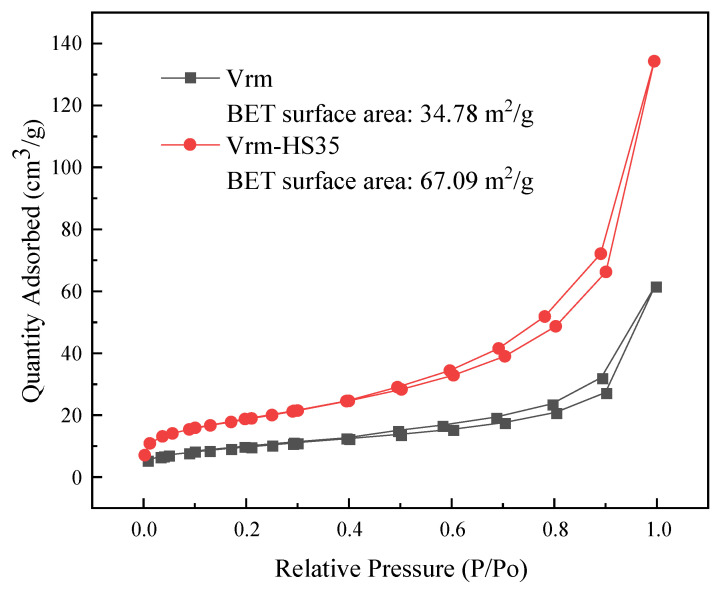
N_2_ adsorption/desorption isotherms of Vrm and Vrm-HS35.

The N_2_ adsorption-desorption isotherms of the fresh and acid-activated vermiculite are presented in Figure 3. As shown in Figure 3, both the adsorption-desorption isotherms overlapped completely at low relative pressures, but a distinct hysteresis loop appeared at high relative pressures, which are similar to type H3 and typical of agglomerates of plate-like particles with slit-shaped pores [37]. After the acid treatment, the specific surface area of Vrm increased significantly from 34.78 m^2^/g to 67.09 m^2^/g, which might be that acid leaching destroyed the crystal structure of Vrm and the micropores formed on the Vrm layer.

### 3.2. Catalytic Properties

#### 3.2.1. Effect of Vermiculite in Cellulose Hydrolysis

Table 1 shows the catalytic results of vermiculite and others reported catalysts for the cellulose hydrolysis in water. From Table 1, the fresh Mt showed the low acidic content and the TRS yield was also lowest. Since the hydrolysis of cellulose is directly correlated to the Brönsted acid sites in aqueous environments, the acid-activated Mt (entry 2) and -SO_3_H grafted solid catalysts (entry 3 and entry 4) showed the higher TRS yield with the higher content of acidic sites than that of Mt. However, comparison to HMt the TRS yield was higher with the relatively low acidic sites on the fresh Vrm catalyst (entry 7). It demonstrated that the content of acidic sites was not the only factor for the hydrolysis of cellulose, and other action had contributed to the conversion of cellulose. Obviously, the acid-activated Vrm catalysts (entry 8 to entry 14) showed the higher TRS yields than that of raw Vrm mineral. Relating it to the BET results, it might be that the acidic sites increased with the increasing of the surface areas after acid treatment and TRS yields improved. Although the acidic sites of the activated Vrm catalysts were lower than that of HMt or Mt-SO3H, the acid-activated Vrm catalysts showed the higher TRS yields. It further proved that another interaction force existed for improving the catalytic properties. Meanwhile, TRS yields of the acid-activated Vrm catalysts (entry 8 to entry 14) displayed the similar variation with that of acidic sites. It also demonstrated that under the same interaction force, the higher content of acidic sites could contribute to the conversion of cellulose to TRS.

**Figure 4:**
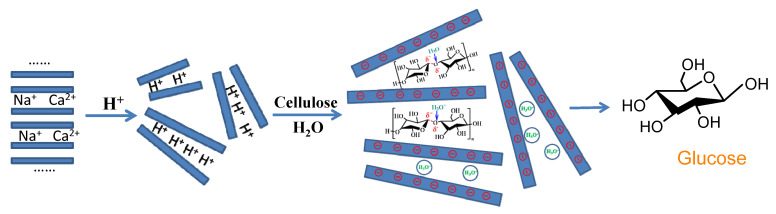
Mechanism of cellulose hydrolysis on acid-activated vermiculite.

It is well-known that Mt and Vrm have similar structures, but Vrm has the higher layer charge density than that of Mt. Moreover, the isomorphic substitution of Vrm mainly occurs in the tetrahedron, while Mt mainly occurs in the octahedron. It demonstrated that the negative charge of Vrm mainly dispersed on the surface and there was higher electrostatic field in the layer of Vrm than that of Mt. So, the high electrostatic action in the layer of Vrm could make the C-O bonds highly polarized and lead to the β-1,4 glycosidic bonds being easily broken. In addition, for the acid-activated Vrm, the content of acidic sites was higher than that of Vrm, which demonstrated that more Brönsted acid sites existed. So, under the synergistic effect of a strongly electrostatic interaction and Brönsted acid, the acid-activated Vrm showed the higher TRS yields than that of raw Vrm and other clay minerals, and the highest TRS yield of 40.1% was obtained on the Vrm treated by 35% H_2_SO_4_ (entry 11). This result demonstrated that the interaction of electrostatic polarization had an important effect on the conversion of cellulose. According to the structure characteristics of Vrm, the possible mechanism of cellulose hydrolysis on the acid-activated Vrm is suggested in Figure 4.

#### 3.2.2. Effect of Reaction Conditions and Reuse of Acid-Activated Vrm in Cellulose Hydrolysis

**Figure 5:**
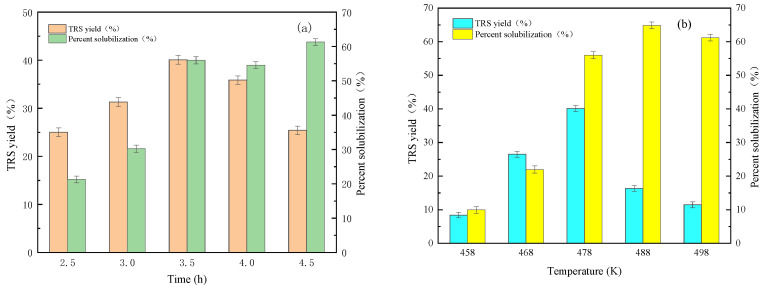

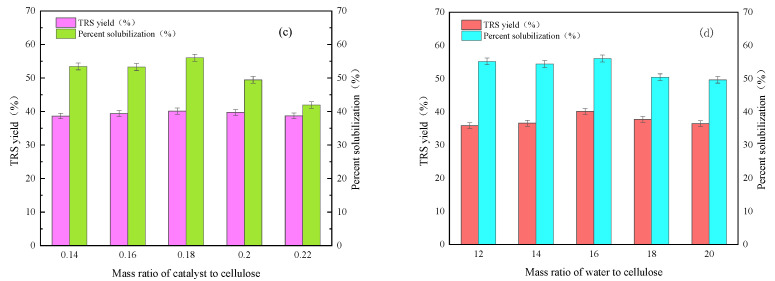
The effect of reaction time (**a**) temperature (**b**) mass ratio of catalysts to cellulose (**c**) and mass ratio of water to cellulose (**d**) on TRS yield and cellulose conversion over Vrm-HS35.

**Figure 6:**
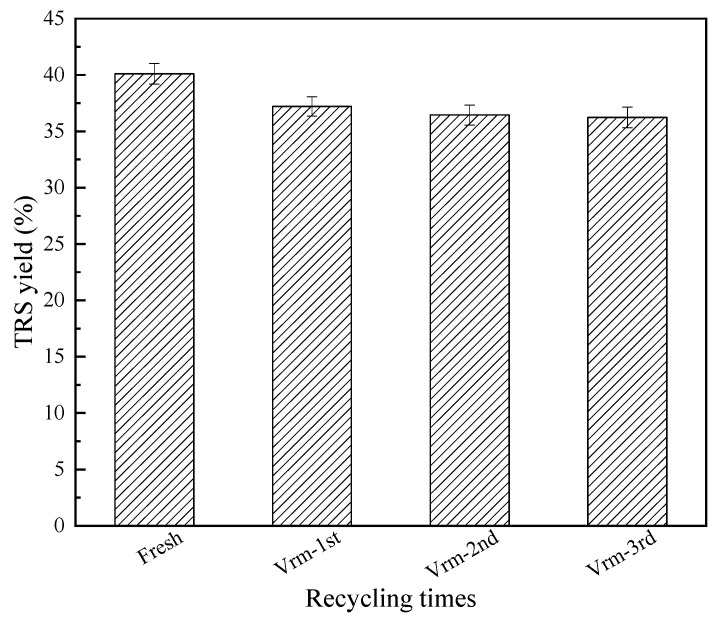
The reuse of acid-activated vermiculite in the cellulose hydrolysis.

It is well known that reducing sugars can be further converted into other products such as levulinic acid, formic acid, or coke. Therefore, the optimal reaction conditions on the hydrolysis of cellulose was further studied. According to our previous work, the parameters were selected and the results are shown in Figure 5. From Figure 5, the optimal reaction time and reaction temperature was 3.5 h and 478 K. This might mean that by-products were easily formed over 3.5 h, and cellulose or reducing sugars were gradually carbonized above 478 K [2,6,9]. As the catalyst/cellulose mass ratio was up to 0.18, and a water/cellulose mass ratio was 16, the highest TRS yield was obtained. It is well known that water is a key factor in the hydrolysis of cellulose, and the hydrogen protons produced by water promote the hydrolysis reaction [7,9]. It should be that there was just the correct concentration of hydrogen protons for the conversion of cellulose in the reaction system under the optimal catalyst/cellulose mass ratio and water/cellulose mass ratio. The concentration of hydrogen protons might be too high in a short time, which would cause the reducing sugars produced to be converted into other by-products or coke. The TRS yield gradually decreased. Therefore, the highest TRS yield of 40.1% could be obtained on the acid-activated vermiculite under the reaction temperature of 478 K, reaction time of 3.5 h, mass ratio of catalyst to cellulose of 0.18 and mass ratio of water to cellulose of 16. The reusability of the recycled catalyst is shown in Figure 6. The TRS yield of each cycle was similar. After three-time reuse, the TRS yield decreased from 40.1% to 36.2%, demonstrating that the acid-treated Vrm was the stable catalyst for the efficient depolymerization of cellulose.

## 4. Conclusions

In summary, acid-activated vermiculite was the efficient and stable catalyst for the hydrolysis of cellulose to RS in water. The vermiculite treated by 35 (wt)% H_2_SO_4_ showed the highest TRS yield of 40.1% among the tested samples under reaction temperature of 478 K, reaction time of 3.5 h, weight ratio of catalyst to cellulose of 0.18, and weight ratio of water to cellulose of 16. After three-time reuse the TRS yield decreased to 36.2%. The crystal structure of vermiculite was almost destroyed and the specific surface area was obviously increased after the acid treatment. However, the high TRS yield on the acid-activated vermiculite should be ascribed to the synergistic effect of electrostatic interaction and Brönsted acid in the interlayer. Considering the low cost, abundance and stability of vermiculite minerals, acid-activated vermiculite catalyst is a promising catalyst for the effective depolymerization of cellulose into reducing sugars.

## Figures and Tables

**Table 1:** The catalytic results of vermiculite and others reported catalysts for the cellulose hydrolysis ^a^.

Entry	Catalysts	Content of Acidic Sites (mmol·g^−1^)	TRS Yield (%)	References
1	Mt	0.012	7.9	[38]
2	HMt	0.32	14.4	[38]
3	Mt-SO_3_H	0.532	24.6	[38]
4	AC-SO_3_H	0.72	21.0	[10]
5	0.3-SZ-Mt	0.03	30.1	[30]
6	Mt-1L	0.056	35.7	[38]
7	Vrm	0.05	17.8	This work
8	Vrm-HS5	0.10	25.9	
9	Vrm-HS15	0.11	28.5	
10	Vrm-HS25	0.13	33.7	
11	Vrm-HS35	0.19	40.1	
12	Vrm-HS40	0.17	38.6	
13	Vrm-HC35	0.13	37.4	
14	Vrm-HP35	0.13	34.6	

^a^ Reaction conditions: microcrystalline cellulose: 0.5 g, catalyst: 0.09 g, water: 8 mL, temperature: 205 °C.

## Sample Availability

Samples of the compounds are available from the authors.
